# Buccal Permeation of Polysaccharide High Molecular Weight Compounds: Effect of Chemical Permeation Enhancers

**DOI:** 10.3390/pharmaceutics15010129

**Published:** 2022-12-30

**Authors:** Adriana Fantini, Luca Giulio, Andrea Delledonne, Silvia Pescina, Cristina Sissa, Sara Nicoli, Patrizia Santi, Cristina Padula

**Affiliations:** 1 ADDRes Lab, Department of Food and Drug, University of Parma, Parco Area delle Scienze 27/a, 43124 Parma, Italy; 2 Department of Chemistry, Life Science and Environmental Sustainability, University of Parma, Parco Area delle Scienze 17/a, 43124 Parma, Italy

**Keywords:** oral mucosa, dextrans, mucosa permeation, chemical enhancer, two-photon microscopy

## Abstract

The greatest achievement in the advanced drug delivery field should be the optimization of non-invasive formulations for the delivery of high molecular weight compounds. Peptides, proteins, and other macromolecules can have poor membrane permeation, principally due to their large molecular weight. The aim of this work was to explore the possibility of administering fluorescently labeled dextrans (molecular weight 4–150 kDa) across the buccal mucosa. Permeation experiments across pig esophageal mucosa were carried out using fatty acids and bile salts as penetration enhancers. The data obtained show that it is possible to increase or promote the mucosa permeation of high molecular weight dextrans by using caprylic acid or sodium taurocholate as the chemical enhancers. With these enhancers, dextrans with molecular weight of 70 and 150 kDa, that in passive conditions did not permeate, could cross the mucosa in detectable amounts. FD-70 and FD-150 showed comparable permeability values, despite the molecular weight difference. The results obtained in the present work suggest that the buccal administration of high molecular weight compounds is feasible.

## 1. Introduction

Macromolecules represent, nowadays, exceptionally promising tools for the treatment of several diseases. The number of macromolecular drugs on the market and in clinical trials is increasing year by year, and the discovery of the molecular basis of the pathologies is expected to bring new high molecular weight (MW) compounds onto the market [1]. In this context, the proteins and antibodies are dominant; however, polysaccharidic compounds are also emerging as bioactive compounds for the treatment of various diseases. For instance, together with the well-known activity of heparin (2–40 kDa) as an anticoagulant, mesoglycan, a mixture of antithrombotic and pro-fibrinolytic glycosaminoglycans, and pentosane sulphate (2–9 kDa) for the treatment of interstitial cystitis, other more recent examples are given by plant-derived polysaccharides with anti-oxidant, immunomodulatory, anti-tumor, anti-inflammatory, anticoagulant, and hypoglycemic activity [2].

Macromolecules often suffer from bioavailability problems linked to low stability and to low permeability across biological membranes. For this reason, at the moment, the preferred administration route is the parenteral one [3]. A possible alternative is represented by the systemic administration of these compounds via buccal delivery. Indeed, the buccal mucosa has been investigated as a potential site for the delivery of macromolecules because of its accessibility [4] and low enzymatic activity compared to the gastro-intestinal tract [5]. The oral cavity contains rich lymphoid tissue, and its pH is favorable for biologics. Moreover, molecules delivered with this administration route avoid the hepatic first-pass effect and degradation by enzymes, such as pepsin, trypsin, and chymotrypsin (which are present in the gastric and intestinal fluids), being absent in the oral mucosa. It should also be noted that the cellular turnover time in the buccal mucosa is slower (4–14 days) than in the gastrointestinal tract, allowing mucoadhesive formulations to adhere to the buccal mucosa for relatively long periods of time. Finally, in the case of adverse reactions, the formulations can be easily and quickly removed, and vomiting and nausea associated with oral administration are avoided. Many low MW drugs have been investigated for oromucosal delivery [6], and also some higher molecular weight molecules, such as the gonadotropin-releasing hormone in dogs [7], octreotide across the buccal porcine mucosa [8], interferon in humans and rodents [9], DNA plasmid in rabbits [10] and insulin both in dogs [11] and in a TR146 cell model of the buccal epithelium in vitro [12]. Additionally, this route could also be used for mucosal vaccination: there is evidence of the ability of oral mucosal vaccine delivery to elicit systemic humoral and cellular immune responses comparable to intramuscular vaccination, enhancing the levels of mucosal antibody responses locally and at distal sites [13].

Despite the potentiality of this administration route, macromolecules are poorly permeable across the buccal mucosa, characterized by a stratified squamous epithelium keratinized or nonkeratinized, depending on the location inside the oral cavity. Indeed, the presence of membrane-coating granules releasing ceramides, acylceramides, and glycosylceramides hinders the permeation of high MW hydrophilic compounds. Previous studies have highlighted that the buccal epithelium has a permeability limit of 20–40 kDa [5], while the sublingual one, owing to the lower thickness, allows for the permeation of molecules as big as 70 kDa [14].

Physical [15,16] and chemical [17] penetration enhancers can represent a valuable tool for promoting the transmucosal flux of macromolecules, thus increasing the possibility of delivering therapeutically relevant amounts of the drug, and also increasing the molecular weight cut-off of the membrane, allowing for the permeation of bigger molecules. Not all substances that alter the barrier integrity can be considered good candidates; in fact, only a small number of chemical enhancers have progressed to clinical testing, having a history of safe use in humans [18]. Among them, we have focused our attention on bile salts, the compounds present in our intestine as a surfactant that emulsifies and solubilizes the agents for fat, and fatty acids, widely used as chemical penetration enhancers in the formulation of the mucosal, dermal, and transdermal dosage forms, to improve drug diffusion.

The aim of this work was to explore the possibility of administering high molecular weight molecules across the buccal mucosa. With respect to previous studies, we have widened the molecules’ MW up to 150 kDa in order to figure out the feasibility of the buccal delivery, even in consideration of the antibodies, antibody fragments, and other high MW compounds. Fluorescently-labeled dextrans (FITC-dextran) of different molecular weights (4 to 150 kDa) were used as the model molecules.

Medium- and long-chain fatty acids and the bile salt sodium taurocholate were tested as penetration enhancers either in pre-treatment or co-administered with FITC dextrans. The porcine esophageal mucosa, an accepted model of the human buccal mucosa [19], was used as a barrier. The structure and lipid composition of the porcine esophageal mucosa are similar to that of the buccal tissue [20], its isolation from the underlying tissues is simpler, its surface is larger, and its integrity is guaranteed.

## 2. Materials and Methods

### 2.1. Materials

Fluorescein isothiocyanate-labeled dextrans of 4 (FD-4), 20 (FD-20), 40 (FD-40), 70 (FD-70), and 150 (FD-150) kDa MWs were purchased from Sigma-Aldrich (St. Louis, MO, USA). Caprylic acid ≥ 99% (C8) and capric acid ≥ 98% (C10) were from Sigma-Aldrich (St. Louis, MO, USA), the lauric acid ≥ 98% (C12) was from Merck (Darmastdt, Germany), and the sodium taurocholate ≥ 95% (NaTC) was from Sigma-Aldrich (St. Louis, MO, USA). All other reagents were of analytical grade.

### 2.2. Methods

#### 2.2.1. Permeation Studies

Ex vivo permeation studies were conducted across the porcine esophageal epithelium. Pig esophagi (Large White or Landrace pigs, both female and male; age: 11–12 months and weight: 145–190 kg) were obtained from a local slaughterhouse (Macello Annoni, Località Madonna dei Prati, Busseto, Italy) within 2 h of the animals’ sacrifice. The esophageal mucosa was separated from the outer muscle layer with a scalpel, and the epithelium was peeled off from the connective tissue after immersion in distilled water at 60 °C for 60 s [21]. Samples obtained were frozen until use, which occurred within 3 months [22]. The tissue was mounted using a regenerated cellulose filter (0.45 µm, pore size) as the inert support in Franz’s type diffusion cells (DISA, Milan, Italy), with a diffusion area of 0.6 cm² and a receptor volume of about 4 mL filled with a degassed pH 7.4 PBS and was kept under magnetic stirring during the experiment.

##### Passive Diffusion Experiments

The donor compartment was filled with 400 µL of an FD solution in PBS pH 7.4 (2 mg/mL, 0.5 mM) without any treatment. Samples of 300 µL were collected from the receptor side at 60 min intervals up to 7 h and replaced with the same amount of fresh PBS.

##### Pre-Treatment with Fatty Acid Permeation Experiments

20 µL of fatty acid—either 50 mg/mL (lauric acid, C12, 250 mM) or 100 mg/mL (caprylic acid and capric, C8 and C10, 690 and 580 mM, respectively)—in an ethanol solution were applied in a non-occluded condition to the epithelium; after 1 h of pre-treatment, the donor compartment was filled with 400 µL of the FD solution (2 mg/mL), as described above “*Passive Diffusion Experiments*”. The diffusion of the permeant was monitored up to 7 h.

##### Co-Administration Permeation Experiments

A donor solution containing NaTC 53.77 mg/mL (100 mM) and FDs (2 mg/mL) in PBS (pH 7.4) was prepared, and 400 µL of this solution was applied to the mucosa.

The duration of the experiments was 7 h.

#### 2.2.2. Analytical Method

The concentration of FDs in the samples was determined using a Spark multimode microplate reader (TECAN, Mannendorf, Switzerland), using 96-multiwell plates flat bottom polystyrene microplates (Corning Life Sciences, Corning, NY, USA). The excitation and emission, λ, were 490 and 520 nm, respectively. A standard curve was developed by plotting different concentrations of the FDs (ng/mL) versus their fluorescence values. The analytical method was validated for specificity (absence of interference from tissue), linearity, and limit of quantification for each dextran.

#### 2.2.3. Data Elaboration

From the linear portion of the permeation profiles, the flux was calculated as the slope of the regression line. Then, the permeability coefficient (P) was obtained using the following relationship:

P = J/C_D_ (1)

where C_D_ (µg/mL) is the donor concentration, and J (µg/cm^−^ h) is the dextrans’ flux across the membrane. The enhancement factor (EF) was calculated as the ratio of the permeability coefficient values in the presence and absence of the penetration enhancer.

The significance of the differences among the results was assessed using a one-way ANOVA followed by a Dunnet’s test. All data are reported as mean ± SEM (*n* = 3–11).

#### 2.2.4. Two Photon Microscopy

Untreated epithelium, as well as tissue left in contact for 4 h with the FD-70 solution after the pre-treatment with caprylic acid, were analyzed with a Two-Photon Microscope, the Nikon A1R MP+ Upright, equipped with a femtosecond pulsed laser Coherent Chameleon Discovery (~100 fs pulse duration, 80 MHz repetition rate, and tunable wavelength output of 660–1320 nm). Specimens were placed into a sample holder and kept hydrated with a saline solution. A 25× water-dipping objective with a numerical aperture 1.1 and 2-mm working distance was employed for focusing the excitation beam and for collecting the two-photon-excited fluorescence (TPEF) signal, which is detected by two non-de-scanned high sensitivity GaAsP detectors. The two detectors are preceded by optical filters, allowing for the simultaneous acquisition of a green channel (506–593 nm) and a red channel (604–679 nm). The imaging overlay of the two channels and processing was performed by the operating software of the microscope. In addition, a fourth GaAsP photomultiplier detector, connected to the microscope through an optical fiber and preceded by a dispersive element, was used to record the spectral profile of the emission signal (wavelength range of 430 to 650 nm with a bandpass of 10 nm). TPEF imaging of all porcine esophagus tissues was collected with an excitation light of 1030 nm, which allowed us to enhance the contrast between the tissue and the fluorescent dextran emissions. The detector gains of the green and red channels were set to the same value for each acquired image.

## 3. Results

The capability of a macromolecule to penetrate a mucosal tissue depends upon its structure and molecular weight, radius, shape, surface charge, and surface polar area. All these properties, in fact, rule the interaction between the macromolecule and the tissue (the epithelia, connective fibers, and extracellular matrix) and can influence partition and diffusion. For this reason, the use of dextrans as the model molecules has notable limitations; while they can mimic relatively well polysaccharide drugs, they lack the hydrophobic domains that are present on the protein surface. Additionally, being neutral molecules, they cannot reproduce the possible ionic interaction taking place between the tissues and charged groups on the protein surface. Despite these limitations, fluorescent-labeled dextrans are widely used to mimic high MW compounds because of their low cost, high analytical sensitivity, and reasonably good predictivity. For instance, they are typically used as markers for blood–brain barrier leakage mimicking proteins [23], as a marker for the effectiveness of skin electroporation [24], to evaluate the capability of the permeation enhancers to improve peptide drug absorption across the intestinal epithelium [25], or to evaluate paracellular transport in oral peptide delivery studies [26].

### 3.1. Permeation Studies across Porcine Esophageal Mucosa: Effect of Dextran Molecular Weight and Comparison with Literature Data

The buccal route represents an interesting approach for high MW compounds’ administration, and ex vivo experiments represent a good starting point for the proof of concept of its feasibility. The model membrane used for this kind of experiment should be, however, very well characterized. In the literature, there is some evidence indicating that the porcine esophageal mucosa is a relevant model of the buccal epithelium [27,28,29,30,31,32,33,34,35,36]. However, most of these studies have been performed with low MW compounds, and these results cannot be directly extrapolated to hydrophilic macromolecules, probably characterized by different penetration pathways [37]. For these reasons, we evaluated the permeability of dextrans (with a MW from 4 to 150 kDa) across the esophageal epithelium and compared the data with literature papers using buccal and sublingual mucosae.

**Figure 1:**
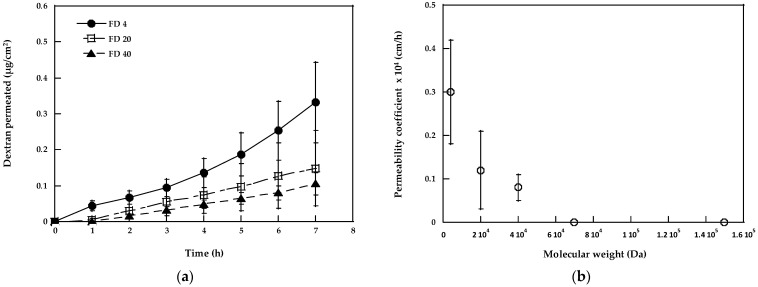
(**a**) Passive permeation profiles of dextrans from 2 mg/mL solutions in the PBS pH 7.4. The amounts of FD-70 and of FD-150 found in the receptor compartment were below the limit of quantification of the analytical method. (**b**) Permeability coefficient of dextrans as a function of molecular weight. Mean values ± SEM (*n* = 5–7).

Figure 1a shows the diffusion profiles of dextrans without any treatment, i.e., in the passive condition, from a 2 mg/mL solution in pH 7.4 PBS.

FD-70 and FD-150 did not cross the membrane in detectable amounts, while the diffusion of lower MW dextrans was roughly in inverse relation to their molecular weight. As expected, the permeability coefficient (Table 1) decreased from 4 k to 40 k Da, as shown in Figure 1b.

The comparison of the data with the freshly excised dermatomed pig buccal mucosa obtained by the other groups (0.40 ± 0.20 × 10^−4^ cm/h [5]) confirms the similarity of the two membranes when a hydrophilic molecule of 4 kDa is evaluated, despite the lower thickness of the esophageal epithelium [19]. The value obtained is slightly smaller compared to the literature data obtained with the sublingual mucosa, 0.72 × 10^−4^ cm/h [14], which is known to be thinner and more permeable than the buccal mucosa. However, despite the higher permeability, sublingual administration is challenging, particularly in view of a sustained release and in terms of locating and retaining the formulation in this region for a long period of time, both for the anatomical conformation and for the presence and stagnation of the salivary fluid that limits the mucoadhesion.

We then compared our data to the literature values concerning the permeability limit; our results highlight an esophageal epithelium permeability limit of 40 kDa, which is intermediate between the freshly excised buccal mucosa (limit of 20–40 kDa) [5] and the sublingual membrane, which is also permeable to FD-70 (with a permeability coefficient of 0.007 × 10^−4^ cm/h [14]).

These differences could be due to the differences in the structure and thickness of the barriers (for instance, the esophageal epithelium has a thickness between 200 and 400 µm, whereas the buccal mucosa has approx. 750 µm [19], and the sublingual epithelium is 100–200 μm [38]), although the differences in the sample preparation (the dermatomed mucosa vs. the isolated epithelium) and/or in the sensitivity of the analytical method might be relevant. Another reason for the difference observed could be linked to the different extent of the tight junctions in the tissues: using laser scanning confocal microscopy, Junginger et al. [5] demonstrated that the paracellular route is the major pathway of dextrans through the buccal mucosa; this penetration pathway has also been confirmed in the intestinal epithelia, and the mechanism involving tight junctions has been identified [8,39].

Despite the above-mentioned differences, the fact that the permeability of the esophageal epithelium toward the dextrans falls within the permeabilities of two regions of the oral cavity can support its use for the study of dextran buccal permeation.

### 3.2. Increasing the Permeability of Macromolecules across the Epithelum

The permeability coefficient found for dextrans is quite small. This, together with the small area available in the oral cavity for permeation, significantly limits the daily dose that can be delivered.

In order to increase the bioavailability of buccally delivered drugs, different approaches can be used, for instance, by increasing the retention time through the mucoadhesive agents or co-administering the enzyme inhibitors [40]. Despite the use of these approaches, the need to increase the permeability of the mucosa to obtain transbuccal fluxes that are clinically significant always remains. For instance, if we consider the treatment of growth failure caused by growth hormone deficiency in children, the usual dose of growth hormone (MWapprox. 22 kDa) is 0.3 mg/kg/week [41] administered by injection (s.c. or i.m.). This dosage is clearly impossible to be administered by the buccal route without a substantial increase in the mucosa’s permeability.

Different kinds of enhancers have been evaluated for peptides and small proteins, such as surfactants (non-ionic, steroidal, and synthetic), bile salts, fatty acids, and chelators [42,43]. In this paper, we have focused attention on fatty acids and bile salts, given their history of safe use. Among the different bile salts, sodium taurocholate was selected. This compound demonstrated the ability to promote insulin delivery across the buccal mucosa in dogs, with a quicker recovery of the mucosal barrier properties in comparison to that of sodium cholate [11]. The lower irritancy of this salt with respect to other bile salts is also highlighted in other mucosal tissues [44], and it is attributed to lower lipophilicity.

#### 3.2.1. Bile Salts

**Figure 2:**
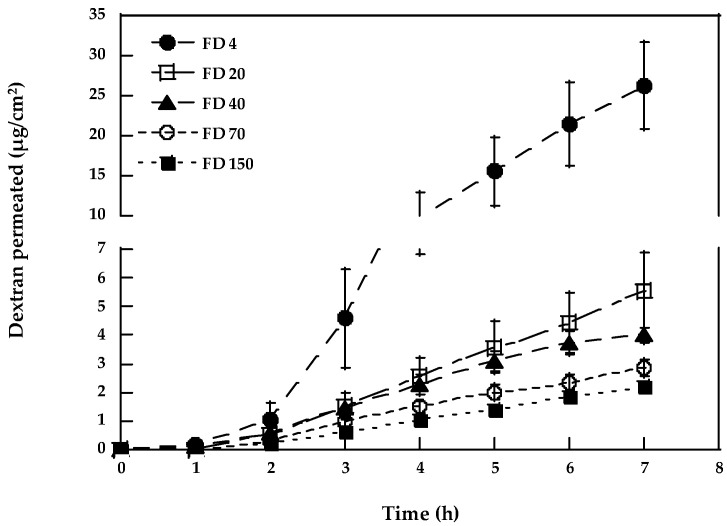
Permeation profiles of dextrans in the presence of 53.77 mg/mL sodium taurocholate (mean values ± SEM).

Figure 2 reports the permeation profiles of the different dextrans in the presence of 53.77 mg/mL of NaTC, and the respective permeation parameters are reported in Table 1, where the enhancement factor (EF) values, calculated as the ratio of the permeability coefficient with and without the enhancer, are also illustrated. A literature paper suggests that the mechanism of action of sodium glycodeoxycholate is dependent on the concentration [45]: at a high concentration (10–100 mM), they act by extracting the cell membrane lipids; a 100 mM concentration was then selected to evaluate the maximum ability of NaTC in the enhancing the diffusion [46,47].

NaTC is able to increase FD-4 transport across the esophageal epithelium by almost two orders of magnitude (EF = 96), whereas the EF obtained for FD-20 and FD-40 is 48 and 49, respectively.

Sodium taurocholate has been demonstrated to increase buccal absorption of different peptides, such as insulin, alpha interferon, and calcitonin [7], as well as to enhance the in vitro and in vivo permeability of the porcine buccal mucosa to fluorescein isothiocyanate-dextran (MW of 4, 10, 20, and 40 kDa) [5]. The enhancement obtained in the present work (see Figure 2 and Table 1) is in reasonable agreement with the data obtained with proteins of similar MWs, such as alpha interferon (MW approx. 20 kDa) [48], whose bioavailability increased 20 times in the presence of 40 mg/mL of NaTC [49].

In addition, by expanding the molecular weight range of the dextrans evaluated, we observed that in the presence of NaTC, the transport of FD-70 and FD-150 was measurable. Since both molecules were unable to cross the esophageal epithelium in passive conditions, it was not possible to calculate their passive fluxes, and for this reason, a statistical analysis of the differences observed was not performed. The permeability coefficient found is higher in the case of FD-70 compared to FD-150 in agreement with the lower MW. The possibility of using a permeation enhancer to allow for the permeation of high MW compounds could, for instance, be exploited in the case of antibodies’ administration or in the case of mucosal vaccination, where virus subunits can have sizes of 70 kDa or bigger [50].

#### 3.2.2. Fatty Acids

In previous work [21], we studied the effect of fatty acids’ pre-treatment on the mucosa penetration of FD-4. In particular, the effect of the concentration, chain length, and the number of double bounds was considered, and from the results obtained, among the fatty acids studied, C8 (applied as a 100 mg/mL solution), C10 (a 100 mg/mL solution), and C12 (a 50 mg/mL solution) resulted as the best performers.

The pre-treatment consisted in the application of a small volume (20 µL) of fatty acid in an ethanol solution in non-occluded conditions for 1 h before the donor compartment was filled with a dextran solution. Because ethanol is a well-known penetration enhancer, the effect of the application of 20 µL of ethanol for 1 h on the dextran permeation was preliminarily examined; the result obtained indicates no difference compared to passive diffusion from an aqueous solution [21].

**Figure 3:**
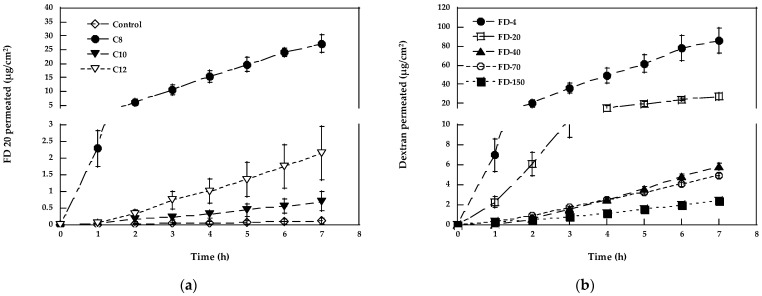
(**a**) Permeation profiles of FD-20 after pre-treatment with different fatty acids; (**b**) permeation profiles of FD-4, FD-20, FD-40, FD-70, and FD-150 after pre-treatment with caprylic acid (C8). Mean values ± SEM.

Figure 3a reports the effect of the pre-treatment with different fatty acids on FD-20 permeation. Among the fatty acids tested, C8 appears as the best performing, followed by C12 and C10, even if, in these last two cases, the permeability increase is not statistically significant due to the high variability.

Given the performance of C8 (100 mg/mL), this fatty acid was tested on the higher molecular weight dextrans. The respective results are reported in Figure 3b and Table 1.

C8 enhances the transport of FD-4, FD-20, and FD-40, with an EF ranging from 219 (FD-4) to 68 (FD-40) and promotes the transport of FD-70 and FD-150, which, in passive conditions, do not cross the mucosa in significant amounts. The permeability coefficients of FD-70 and FD-150 were very similar, indicating that the enhancing effect was comparable despite the large difference in the molecular weight of the permeant. The mechanism of action proposed for the fatty acids includes increased partitioning, the extraction of the intercellular lipids, membrane destabilization due to cholesterol dissolution, a change of membrane fluidity, and a disturbance of the lipid packing [17,51]. According to [52], fatty acids may interact with the phospholipids of cell membranes, increasing drug diffusion via the non-polar route. In another report, the opening of the tight junction for macromolecules up to a certain molecular weight has been hypothesized to explain the effect of sodium caprate on dextrans’ permeation across cultured intestinal cells [53]; it has been also suggested that sodium laurate can open the tight junction by the reversible relocation of Claudin-5 [54]. Then, the absence of difference between FD 70 and FD 150 may indicate the opening of tight junctions to molecules as big as FD-150 [55].

**Figure 4:**
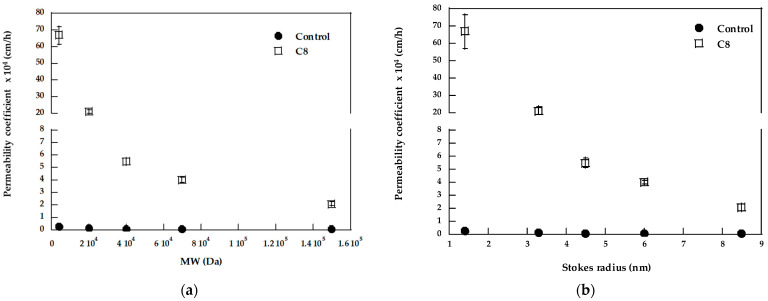
Permeability coefficient of dextrans as a function of molecular weight (**a**) and of Stokes radius (**b**). Stokes radius was from the Supplier (https://www.sigmaaldrich.com/deepweb/assets/sigmaaldrich/product/documents/425/562/fd70pis.pdf, accessed on 15 October 2022). Mean values ± SEM.

The effect of fatty acids on the dextran permeability coefficient is summarized in Figure 4, where it is plotted as a function of the molecular weight (panel a) and Stokes’ radius (panel b), a more comprehensive parameter that takes into account also shape and compactness [14].

From Figure 4, it is evident that for lower MW dextrans (FD-4 and FD-20), the pre-treatment with C8 is much more effective for comparison with dextrans with a higher molecular weight. This result suggests a different penetration mechanism of the dextrans tested; we can hypothesize that higher MW dextrans enter the tissue only by the paracellular route following the tight junction opening. In these conditions, the diameter of the pore pathway has been estimated to be around 100 Å [56]. Lower MW dextrans can penetrate the tissue also by the intracellular route, and, for this reason, they are also sensitive to changes in the membrane’s fluidity and disturbance of the lipid packing. This justifies the higher EF found since they can take advantage of the two different enhancing mechanisms.

To further investigate the mechanism of the penetration of FD-70 across the epithelium and the effect of the caprylic acid pre-treatment, two-photon microscopy was used.

### 3.3. Two-Photon Microscopy

To the best of our knowledge, the literature does not report any description of the porcine esophageal mucosa obtained by two-photon microscopy. Two-photon microscopy is an innovative technique allowing for three-dimensional and deep tissue imaging without sample fixation, sectioning, and staining. In comparison to confocal fluorescence microscopy, two-photon microscopy works with less energetic photons (near infrared excitation), thus reducing the photodamage caused by UV and visible light and increasing the penetration depth [57].

**Figure 5:**
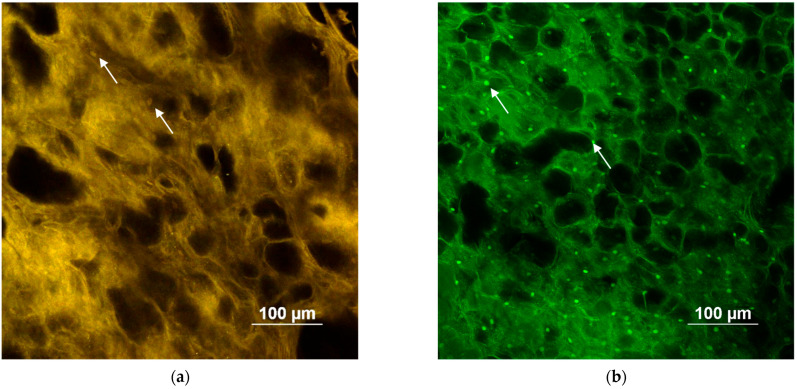

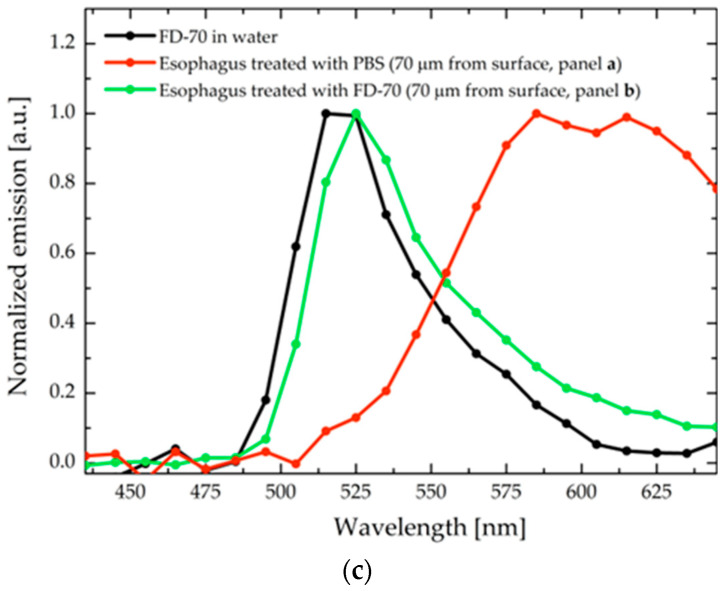
(**a**,**b**): TPEF images (image size: 500 × 500 μm) acquired at an intermediate depth (~70 μm from the surface) with an excitation wavelength of 1030 nm from the blank and treated with caprylic acid (C8) tissues, respectively; (**c**) comparison between the normalized emission spectra of an FD-70 aqueous solution and the blank and treated tissues (excited at 1030 nm and recorded in correspondence with the (**a**,**b**) focal plane). Arrows in panels **a** and **b** display epithelial cell nuclei.

The tissues were simply pre-treated for 1 h with 100 mg/mL of caprylic acid [21] and left in contact with the FD-70 solution for 4 h (test). All the samples were washed with water and visualized. Figure 5 compares the test and untreated samples, highlighting the substantial penetration of FD-70 in the tissue depth. The test sample is characterized by a green emission, which is ascribable to the emission of FITC, as confirmed by the emission spectrum (Panel c). On the contrary, the autofluorescence of the reference tissue is detected both in the green and red channels, giving rise to a yellowish image (Panel a). In both tissues, some epithelial cells can be recognized. The treatment with FD-70 uniformly stains the tissue and facilitates the identification of cell nuclei (the white arrows in Panel b), which is less visible in the reference sample (the white arrows in Panel a). In principle, such behavior is compatible with a combination of the paracellular and intracellular routes for the penetration of FD-70. The emission spectra recorded (Panel c) confirm the different emission profiles for the two samples, with the treated one having a peak superimposable with the FD-70 in solution.

**Figure 6:**
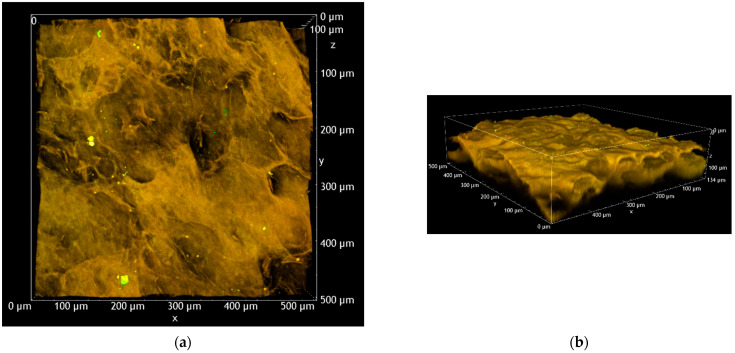

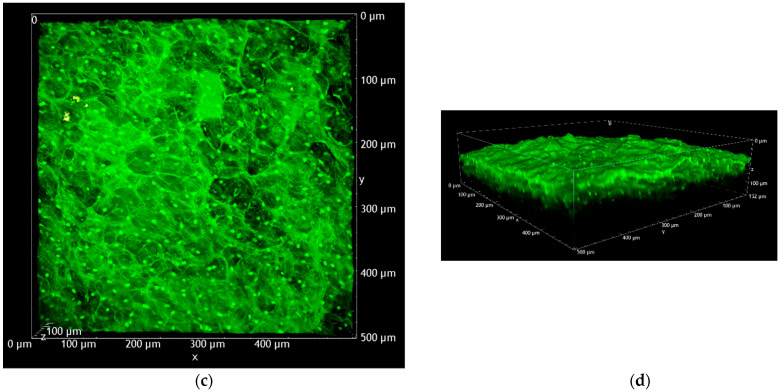
Volume renderings of the pre-treated with caprylic acid (C8) porcine esophageal epithelium, reconstructed from the Z-stack. Blank tissue: (**a**) XY view; (**b**) 3D overview. Test tissue treated with FD-70: (**c**) XY view; (**d**) 3D overview. All the images were acquired with an excitation wavelength of 1030 nm and equal detector gains in the green and red channels.

The apparent presence of “holes” in the tissue can be attributed to the corrugated surface of the mucosa, as can be revealed from the volume renderings reported in Figure 6 and Supplementary Videos S1 and S2. From Figure 6, it is possible to safely assess that, after 4 h of permeation, FD-70 has fully stained the first 130 μm of the esophageal tissue. Most probably, the underlying part of the tissue also retained the fluorescent dextran, but due to the high scattering of the samples, it was not possible to acquire images in the deeper regions.

## 4. Conclusions

The data reported in the present paper show that it is possible to increase or promote the mucosa permeation of high molecular weight dextrans, used as the model molecules, by using sodium taurocholate or, better, caprylic acid as chemical enhancers. We have demonstrated, for the first time, that with these enhancers, used in co-administration or as a pre-treatment, respectively, dextrans with a molecular weight of 70 and 150 kDa could cross the mucosa in significant amounts. It must, however, be underlined that at this stage, it is difficult to figure out if the amount permeated can be enough to reach a therapeutic goal. This is, in fact, strictly linked to the potency of the compound and its eventual metabolization in vivo. Another limitation of the present study is represented by the lack of toxicity studies since the histology of the tissue was not evaluated to figure out possible membrane changes. It is, however, important to underline that the effect on the mucosa is expected to be transient and that the rapid turnover of the buccal tissue will allow a rapid recovery of the barrier function [11]. This topic, however, surely deserves further studies.

Finally, some comments on the administration mode should be given. The present study represents the proof of concept of the feasibility of the buccal delivery of high MW compounds; it is, however, clear that buccal administration requires a complex formulation, able to retain the active compound in contact with the membrane for a long enough time period. This could be done, for instance, by using mucoadhesive hydrogels co-loaded with the drug and enhancer. In the case of the pre-treatment, the in vivo application might be challenging. Furthermore, standardized application protocols should be established, combining the enhancer formulation with the drug formulation.

## Figures and Tables

**Table 1:** Permeation parameters of dextran across the pig esophageal epithelium (mean values ± SEM) using fatty acids and bile salts as penetration enhancers.

Dextran	Enhancer	Administration Mode ^a^	Flux (µg/cm^2^ h)	Permeability Coefficient × 10^−4^ (cm/h)	EF	Number of Replicates ^c^
FD-4	None	-	0.06 ± 0.02	0.30 ± 0.12	-	7
	C8	Pre-treatment	13.36 ± 1.96 ***	66.78 ± 9.78	219	5
	NaTC	Co-administration	5.84 ± 1.01 ***	29.21 ± 5.06	96	7
FD-20	None	-	0.02 ± 0.01	0.12 ± 0.09	-	6
	C8	Pre-treatment	4.24 ± 0.47 ***	21.22 ± 2.37	171	6
	C10	Pre-treatment	0.13 ± 0.06	0.64 ± 0.29	5	3
	C12	Pre-treatment	0.39 ± 0.14	1.93 ± 0.69	16	11
	NaTC	Co-administration	1.20 ± 0.21 ***	5.99 ± 1.34	48	6
FD-40	None	-	0.016 ± 0.003	0.08 ± 0.03	-	7
	C8	Pre-treatment	1.10 ± 0.07 ***	5.49 ± 0.35	68	6
	NaTC	Co-administration	0.8 ± 0.05 **	4.00 ± 0.26	49	11
FD-70	None	-	n.d. ^b^	-	-	4
	C8	Pre-treatment	0.80 ± 0.03	3.99 ± 0.13	-	4
	NaTC	Co-administration	0.46 ± 0.04	2.29 ± 0.22	-	9
FD-150	None	-	n.d. ^b^	-	-	6
	C8	Pre-treatment	0.59 ± 0.06	2.95 ± 0.29	-	4
	NaTC	Co-administration	0.40 ± 0.03	2.01 ± 0.15	-	8

^a^ Pre-treatment was performed by applying 20 µL of fatty acid—either 50 mg/mL (lauric acid, C12) or 100 mg/mL (caprylic and capric acid, C8 and C10)—in an ethanol solution for 1 h in non-occlusive conditions. ^b^ The concentrations found were below the limit of quantification of the analytical method. ^c^ The high number of replicates is linked to the high variability of the permeation data. Permeation across the buccal mucosa is also characterized by high variability [37]. ** *p* < 0.01 and *** *p* < 0.001 are significantly different from the respective passive condition.

## Data Availability

Data are available on motivated request.

## Supplementary Materials

The following supporting information can be downloaded at: https://www.mdpi.com/article/10.3390/pharmaceutics15010129/s1, Video S1: 3D reconstruction of untreated porcine mucosa (excitation wavelength 1030), Video S2.: 3D reconstruction of porcine mucosa pre-treated with caprylic acid, after 4 h contact with FD-70 solution (excitation wavelength 1030).
